# Application of PCR-ELISA in Molecular Diagnosis

**DOI:** 10.1155/2014/653014

**Published:** 2014-05-27

**Authors:** Mei Jean Sue, Swee Keong Yeap, Abdul Rahman Omar, Sheau Wei Tan

**Affiliations:** Laboratory of Vaccines and Immunotherapeutics, Institute of Bioscience, Universiti Putra Malaysia (UPM), 43400 Serdang, Selangor, Malaysia

## Abstract

Polymerase chain reaction-enzyme linked immunosorbent assay (PCR-ELISA) is an immunodetection method that can quantify PCR product directly after immobilization of biotinylated DNA on a microplate. This method, which detects nucleic acid instead of protein, is a much more sensitive method compared to conventional PCR method, with shorter analytical time and lower detection limit. Its high specificity and sensitivity, together with its semiquantitative ability, give it a huge potential to serve as a powerful detection tool in various industries such as medical, veterinary, and agricultural industries. With the recent advances in PCR-ELISA, it is envisaged that the assay is more widely recognized for its fast and sensitive detection limit which could improve overall diagnostic time and quality.

## 1. Introduction

In the early 90s, there was a sudden interest in DNA studies when Friedrich Miescher first identified and isolated DNA and when James D. Watson and Francis Crick first discovered the double helix structure of DNA in 1953. From then on, various molecular techniques and knowledge were introduced such as gel electrophoresis, DNA double helix structure, and the invention of polymerase chain reaction (PCR) by Kary Mullis in 1983, one of the most innovative and still widely used techniques in the field of  life sciences. Although PCR is a powerful tool, its applications cannot be fully expressed without a powerful detection tool.

Gel electrophoresis is one of the commonly used methods for the detection of an amplified PCR product but this method has a low detection limit and only allows the user to detect the presence or absence of a particular gene. Many detection methods and equipment have since been developed and amongst those commonly used is real-time PCR. In the late 1980s, there was a sudden boom of interest in the study of immunodetection of DNA. Various methods of immunodetection were published and amongst them is a study by Coutlée et al. [1] where they studied the immunodetection of DNA using biotinylated RNA probes. From then on, numerous studies on immunodetection of DNA using enzyme linked immunosorbent assay techniques were published, which subsequently lead to the introduction of polymerase chain reaction-enzyme linked immunosorbent assay (PCR-ELISA). This method combines both PCR and ELISA into a single analytical technique and its application is very much similar to ELISA except that this method allows the detection of nucleic acid instead of protein [2].

**Figure 1:**
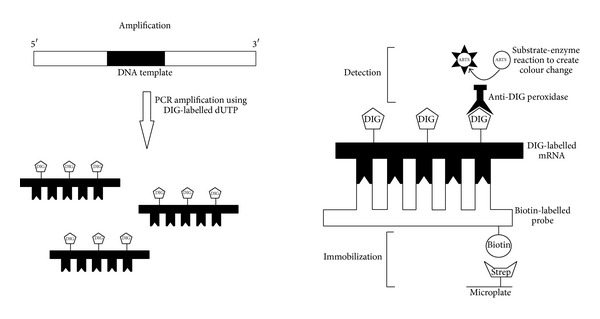
Illustration of the 3-step PCR-ELISA method: (i) amplification of the gene of interest using PCR in the presence of DIG-dUTP, which is then bound to specific probes, (ii) immobilization of the gene of interest to the microplate through strong affinity of avidin-biotin interaction, followed by (iii) detection of biotinylated DNA using an anti-DIG-peroxidase conjugate with substrate ABTS to form a blue-green color reaction that is both visible and measured using a spectrophotometer.

How does PCR-ELISA work? PCR-ELISA is an immunological method to quantify the PCR product directly after immobilization of biotinylated DNA on a microplate. The whole method involves 3 steps: amplification, immobilization, and detection. At the very beginning of the method, the gene of interest will be amplified through PCR in the presence of digoxigenin-11-dUTP (DIG-dUTP). DIG-labelled PCR products will then bind to specific oligonucleotide probes, labelled with biotin at their 5′ end. The next step involves immobilizing the gene of interest to the microplate. This is achievable with the presence of streptavidin coated on microplates and biotin on the 5′ end of the formed hybrid. Strong affinity of avidin-biotin interaction forms the avidin-biotin complex, thus binding only PCR products with the specific gene of interest to the microplate. All other nonspecific products will be washed off. After immobilization, detection of biotinylated DNA is required as the formation of these complexes cannot be detected through naked eyes. To do so, the amplicons can be detected using an anti-DIG-peroxidase conjugate through the substrate 2,2′-azino-di-3-ethylbenzthiazoline sulfonate (ABTS). These will develop a blue-green color reaction that is both visible and measured using a spectrophotometer (Figure 1) [3]. Another method of PCR-ELISA detection includes the use of fluorescein probe where detection includes the use of antifluorescein antibodies conjugated to horseradish peroxidase to detect the hybridized fluorescein-labelled oligonucleotide probe [4].

## 2. Comparisons of PCR-ELISA with Other PCR-Based Molecular Approaches 

Since the introduction of this tool, various studies have been carried out to compare the performance of PCR-ELISA with other tools. Many agreed that the detection of DIG-labelled products by microwell capture hybridization assay makes PCR-ELISA a more sensitive tool than agarose gel electrophoresis analysis because the specific hybridization and enzymatic colourization increase the positive signal of biotin-labelled, probe-bound PCR products.

The PCR amplicons are analyzed using a colorimetric assay; thus not only is there reduced risk on the use of mutagen-staining materials and significant reduction of possible DNA contamination, but also it allows the method to serve as a semiquantitative tool [5]. As this detection uses gene-specific probes for detection, the specificity of the tool is very prominent [2, 6]. Not only can samples with that particular gene be detected, but also they can be quantified based on colour intensity. The presence of higher colour intensity indicates that more probe was bound to the specific gene sequence, forming a hybrid complex that was later bound by a peroxidase-conjugated anti-DIG antibody and the colorimetric peroxidase substrate ABTS, allowing detection. Whilst PCR-ELISA cannot provide an accurate estimation of the actual gene of interest that is present compared to real-time PCR (qPCR), it provides a quick summary of whether a particular substrate is high or low at a particular time, as colorimetric detection is in direct proportion to the number of the intended gene of interest.

Another main attraction of PCR-ELISA is that the assay allows large-scale screening to be done using only standard laboratory equipment, making it suitable to be used in clinical laboratories. This should serve as another incentive for laboratories with fewer resources as a survey done by Comley [7] showed that respondents do not turn to fully automated equipment for ELISA despite its availability, possibly due to the high costs of purchasing and maintaining the equipment. Last but not least, the overall analytical time of this assay is also much shorter than conventional PCR method, making it a promising tool for future uses especially when dealing with a large sample size.

With new discoveries and new invention, molecular biology tools need to be continuously improved and developed for faster and more efficient results. Each new technology that was developed has its pros and cons compared to other technologies. If the study is about an unknown gene, then conventional PCR using agarose gel detection would be the only available option as both qPCR and PCR-ELISA require the development of primers and/or probes that is difficult to achieve on a new target gene.

There were several articles comparing PCR-ELISA with qPCR for their high sensitivity ability. Menotti et al. [8] who did a comparison between PCR-ELISA and qPCR assay for the detection of* Toxoplasma gondii*, a parasitic protozoan that is responsible for causing life-threatening infections in immunocompromised hosts, found that the former method yields negative results in 15 samples that were clinically proven to suffer from the disease while the latter presented accurate results throughout the study. This was supported by various other authors (Table 1) whereby qPCR proved to be a more sensitive tool. Without the presence of any competitor so far in terms of sensitivity, qPCR is still an essential tool in research studies for detection. However, apart from this handicap, PCR-ELISA was found to be more cost effective as real-time PCR requires the use of costly fluorescent scoring system. If the purpose of the study does not require such high sensitivity as compared to qPCR, PCR-ELISA might just be the better option as it offers semiquantitative ability and adequate sensitivity at lower costs [9].

Overall, the main factors that need to be considered while choosing the best method for their experimental design would be the pricing factor and level of sensitivity required. Several studies compared the use of qPCR, PCR-ELISA, and conventional PCR for the detection of poultry virus, infectious bursal disease virus, and the summary of the results is listed in Table 1 [2, 10–14].

## 3. Applications of PCR-ELISA

With the aforementioned advantages of PCR-ELISA and its semiquantitative ability, a number of researchers propose the use of PCR-ELISA in a diverse range of fields, from basic detection and diagnosis to quality control and quantitative monitoring of infectious disease, food allergen detection, plant pathogens and biomarkers, with detection as its main application.

### 3.1. Detection and Diagnosis

Due to its high sensitivity and specificity, various studies on the use of PCR-ELISA as a detection and diagnostic method were proven successful. As rapid diagnosis in the medical field can affect the life and death of the public, the papers below are amongst some of the recent studies reported within the last 5 years on the detection of various diseases and pathogens in the medical diagnosis: identification of cancer cells [15–17]; detection of the presence of Hepatitis A, B, C, and E types [14, 18–21]; species detection and identification of dermatophyte species [22]; invasive fungal infections in immunocompromised patients [23–25]; detection of poliovirus, enterovirus, and norovirus [26, 27]; and determination of blood group antigens for hemolytic disease of the newborn cases and polytransfused patients [28].

There are also a number of publications using PCR-ELISA in the food industry such as the detection of harmful food-borne pathogens such as* Campylobacter* sp.,* Salmonella* [6, 29–32],* Listeria monocytogenes* [33, 34],* Escherichia coli* [26, 35],* Brucella melitensis* [36], and* Vibrio parahaemolyticus* [2]. Not limiting the use of the method in the medical and food industries, the study of PCR-ELISA extends even to the veterinary industry. Amongst the studies are* Leishmania *parasite detection [37, 38] and the detection of various avian viruses in chickens [10, 39, 40]. Other detection studies that help detect the presence of plant pathogens [41, 42] include tomato spotted wilt [43], potato spindle tuber [12], prevalence of each phylogenetic group among the infected grapevine varieties [44], and plant viruses in woody plants [45]. PCR-ELISA can also detect the presence of harmful waterborne pathogens in both water supply and industrial cooling tower water [46, 47].

As PCR-ELISA is a sensitive tool that allows detection at very small concentration, there are suggestions to develop PCR-ELISA as an early detection system that allows preventive measures to be taken before the condition of the patient deteriorates or the situation worsens. The use of PCR-ELISA as an early warning system can be extended for the detection of latent symptoms of diseases or even gene expression studies through biomarkers. Some of the studies published include the development of PCR-ELISA as a replacement method for detection and validation of gene expression studies [9] and detection of four *β*-thalassemia point mutations in Iranians using a PCR-ELISA genotyping system [5].

### 3.2. Quantitative Monitoring

Many studies also suggest the use of the assay for quantitative monitoring as a quick indication in the presence or absence of a particular substrate and its estimated concentration. It is a very crucial tool, especially for immunocompromised patients who are sensitive and susceptible to their environment, as it allows the determination of appropriate level of antiviral management. Amongst studies on the quantification monitoring using PCR-ELISA are evaluation and monitoring of cytomegalovirus infection in bone marrow transplant recipients [48], diagnostic value of the combined determination of telomerase activity in induced sputum, pleural effusion, and fibrobronchoscopic biopsy in lung cancer patients [49], and quantitative monitoring of* Leishmania *parasite in livestock [37].

## 4. Recent Advances in PCR-ELISA Technologies

Due to its ability to test multiple samples in a single run with shorter running time, a number of PCR-ELISA trial runs are currently in progress for use in medical diagnosis. With the success of these trial runs, it can be foreseen that the assay would be used in various quality control and medical diagnostic labs in the near future. Even with its high sensitivity and specificity, there are continuous attempts to improve the applications of PCR-ELISA in recent years. As PCR protocols are already established, the focal point for further improvements is on the immunoassay detection of DIG-labelled PCR products, such as the effects of streptavidin concentration on the microplate, addition of various PCR products to the microplate, or other solid-phase interfaces which require thorough optimization [6].

One of recent attempts to improve PCR-ELISA is to develop multiplexing ability, where several specific sequences are detected simultaneously, without any cross-reactivity. This method is designed as many felt that separate reactions for each species are both costly and time consuming compared to simultaneous detection. The key development for this procedure is to design probes that are highly specific for each of the species of interest but with the absence of cross-reactions [11, 13, 18, 50–55].

Kobets et al. [37] developed an improved and optimized PCR-ELISA method that eliminates the need for a separate step of hybridization of the PCR product with labelled probes. Focusing on the detection and quantification of* Leishmania *parasites, these researchers use both DIG- and biotin-labelled primers to produce PCR products. These products were then attached to the streptavidin-coated plate before the products were detected using sandwich ELISA with anti-DIG antibodies. Not only does this method eliminate the hybridization step but also it eliminates a number of steps, including all the washing procedures in between each step. Amongst the procedures that were also eliminated are the denaturation step of PCR product prior to hybridization and the need for designing and attaching specific probe to the microplate. Overall, this design reduces the incubation and washing time, as well as reagent costs.

Others developed a technique that eliminates the need to denature and neutralize samples prior to hybridization compared to the conventional PCR-ELISA method. This method, known as asymmetric PCR-ELISA, utilizes asymmetric PCR amplification to amplify only one DNA strand in a double-stranded DNA template using excess primer for the strand targeted for amplification prior to detection. Nolasco et al. [41, 42] found that asymmetric PCR-ELISA increases the relative concentration of the target DNA species, making this method more sensitive than TaqMan detection method. The method also utilizes less dNTPs and DIG labels, which are very costly, by half and tenfold, respectively, without reducing its sensitivity.

No studies have yet been attempted to compare these few methods to identify the advantages and disadvantages of each method but it seems that the advances seem to be focusing on reducing the production costs and turn-round time whilst maintaining the assay's sensitivity and specificity.

## 5. Summary

Overall, PCR-ELISA method (i) is much more sensitive by about 100-fold than conventional PCR method, (ii) is with short analytical testing time, allowing faster result output, (iii) allows multiple sample testing with the use of gene-specific probes, (iv) is able to do quantitative and qualitative analyses, (v) reduces risk of contamination, (vi) omits the use of mutagen-staining materials, and (vii) is an easy-to-use method as it only requires the use of basic lab equipment. By offering faster diagnostic time and high sensitivity, there is high potential for PCR-ELISA to serve as a powerful detection tool in all sectors, from medical sector to both food and agriculture sectors. Early detection allows earlier management intervention, allowing more lives to be saved, overcoming food shortages problem indirectly, and preventing contaminated food products from reaching the consumers. Since a number of trial runs on the tool are already ongoing, one can hope that the diagnostic time and quality will be further improved in the near future.

## Figures and Tables

**Table 1:** Comparisons between 3 different detection methods; conventional PCR with agarose gel electrophoresis, PCR-ELISA and qPCR.

Comparison	Conventional PCR	PCR-ELISA	qPCR
Equipment required	Standard laboratory equipment	Standard laboratory equipment	Requires fluorescence detection instrument
Reagent costs	Low	Moderate	Costly
Detection limit	1–10 ng/*μ*L	0.01 ng/*μ*L	0.25 pg/*μ*L
Quantitative ability	Not quantitative	Semi-quantitative	Quantitative
